# Evaluation of a single round polymerase chain reaction assay using dried blood spots for diagnosis of HIV-1 infection in infants in an African setting

**DOI:** 10.1186/1471-2431-11-18

**Published:** 2011-02-18

**Authors:** Bhavna H Chohan, Sandra Emery, Dalton Wamalwa, Grace John-Stewart, Maxwel Majiwa, Musa Ng'ayo, Steve Froggett, Julie Overbaugh

**Affiliations:** 1 Department of Medical Microbiology, University of Nairobi - College of Health Sciences (off Ngong Road), Nairobi (Box 19767-00202), Kenya; 2 Department of Medicine, Division of Allergy and Infectious Diseases, University of Washington (Pacific Street), Seattle (98104), WA, USA; 3 Division of Human Biology, Fred Hutchinson Cancer Research Center (1100 Fairview Ave N.), Seattle (98104), WA, USA; 4 Department of Paediatrics, University of Nairobi - College of Health Sciences (off Ngong Road), Nairobi (Box 19767-00202), Kenya; 5 Department of Epidemiology, University of Washington (325 9thAve), Seattle (98104), WA, USA; 6 Center for Microbiology Research, Kenya Medical Research Institute (Hospital Street), Nairobi (Box 19464-00202), Kenya

## Abstract

**Background:**

The aim of this study was to develop an economical 'in-house' single round polymerase chain reaction (PCR) assay using filter paper-dried blood spots (FP-DBS) for early infant HIV-1 diagnosis and to evaluate its performance in an African setting.

**Methods:**

An 'in-house' single round PCR assay that targets conserved regions in the HIV-1 polymerase (*pol*) gene was validated for use with FP-DBS; first we validated this assay using FP-DBS spiked with cell standards of known HIV-1 copy numbers. Next, we validated the assay by testing the archived FP-DBS (N = 115) from infants of known HIV-1 infection status. Subsequently this 'in-house' HIV-1 *pol *PCR FP-DBS assay was then established in Nairobi, Kenya for further evaluation on freshly collected FP-DBS (N = 186) from infants, and compared with findings from a reference laboratory using the Roche Amplicor^® ^HIV-1 DNA Test, version 1.5 assay.

**Results:**

The HIV-1 *pol *PCR FP-DBS assay could detect one HIV-1 proviral copy in 38.7% of tests, 2 copies in 46.9% of tests, 5 copies in 72.5% of tests and 10 copies in 98.1% of tests performed with spiked samples. Using the archived FP-DBS samples from infants of known infection status, this assay was 92.8% sensitive and 98.3% specific for HIV-1 infant diagnosis. Using 186 FP-DBS collected from infants recently defined as HIV-1 positive using the commercially available Roche Amplicor v1.5 assay, 178 FP-DBS tested positive by this 'in-house' single-round HIV-1 *pol *PCR FP-DBS PCR assay. Upon subsequent retesting, the 8 infant FP-DBS samples that were discordant were confirmed as HIV-1 negative by both assays using a second blood sample.

**Conclusions:**

HIV-1 was detected with high sensitivity and specificity using both archived and more recently collected samples. This suggests that this 'in-house' HIV-1 *pol *FP-DBS PCR assay can provide an alternative cost-effective, reliable and rapid method for early detection of HIV-1 infection in infants.

## Background

Although interventions to prevent mother-to-child transmission of HIV-1 infection are increasingly implemented as part of national guidelines, the prevalence of pediatric HIV-1 infection remains high in Africa. It is projected that about 1000 new pediatric cases occur daily worldwide, with 90% occurring in sub-Saharan African countries [1,2]. Hence, an accurate economical and reliable early infant diagnosis of HIV-1 infection in Africa has become of paramount importance as such diagnosis can ensure that antiretroviral therapy is promptly provided for those in need. In addition infant HIV-1 diagnosis is the best measure for evaluation of mother-to-child transmission programs and can facilitate appropriate stratification of healthcare services [3].

Molecular methods such as polymerase chain reaction (PCR) assays are the most sensitive method for infant HIV-1 diagnosis [3-10] because passively acquired maternal antibodies in the infant complicates the use of conventional HIV-1 serologic diagnostic assays. Currently, a variety of validated commercially available and 'in-house' PCR-based methods that detect HIV-1 nucleic acids are available [3,5-8,10-13]. Many of these methods have been adapted for HIV-1 diagnosis using either whole blood, or dried blood spots collected on filter papers (FP-DBS), which are more convenient for collection, transport and storage. However many of these commercial PCR-based assays on FP-DBS for early HIV-1 infant diagnosis are expensive (in the range of $20-$50 per assay), and therefore beyond the reach of the majority of the population that resides in low-resource settings where the epidemic is prevalent [3]. Hence, there has been an urgent need for cheaper and reliable assays for early HIV-1 infant diagnosis.

Previously, our laboratory evaluated an 'in-house' PCR assay for HIV diagnosis that relied on a two round, nested PCR amplification of the HIV-1-*gag *sequences from FP-DBS [4]. The PCR results using FP-DBS showed 100% specificity, and 96% sensitivity (based on quadruplicate testing) compared to results with blood mononuclear cells collected from paired venous blood [4]. However, an assay that relies on two rounds of PCR can be challenging in laboratories that do not have optimal facilities for minimizing PCR contamination.

Here we describe an inexpensive single round PCR that requires minimal nucleic acid manipulation and compare its performance with the earlier HIV-1-*gag *PCR assay and the commercial Roche qualitative HIV Amplicor^® ^DNA PCR, version 1.5 assay, which is currently the assay with extensive validation in Africa [3].

## Methods

### PCR methods

The PCR method described here was a modification of a previously described real-time PCR assay that targets the HIV polymerase (*pol*) gene [14,15]. Minor changes were made by shifting the primers to minimize non-specific amplification. The primers used were forward primer pol 151 5'TACAGTGCAGGGGAAAGAATAATAG3' (corresponds to positions 4809 - 4833 in HXB2) and the reverse primer pol 40 5'CTACTGCCCCTTCACCTTTCC3' (position 4954- 4974 in HXB2). The PCR reaction mixture contained 150 μmol/L of MgCl_2_, 200 μmol/L of dNTP, 1 μmol/L of each primer, 1.5U of ABI AmpliTaq Gold Polymerase and appropriate buffer mix (Applied Biosystems), 0.1% of Bovine Serum Albumin, and 2 μl of the DNA template. The cycling parameters used were 50°C for 2 min; 95°C for 10 min, 1 cycle; 95°C for 15s and 60°C for 42 cycles. The expected product is 166bp, which was visualized by gel electrophoresis through 2% agarose and ethidium bromide staining. We refer to this assay as the HIV-1 *pol *PCR FP-DBS assay.

### Extraction of nucleic acids from FP-DBS

The nucleic acids were extracted from the FP-DBS by two different methods, depending on the assay performed on the sample.

For the regular 'in-house' *pol *and *gag *PCR FP-DBS assay, a lysate was prepared by lysing the blood sample from the FP-DBS, using an ethanol-flamed 8mm hole punch to detach a blood spot, which samples about one quarter of the total blood spot. Nucleic acids from the DBS spot were extracted using a quick lysis approach, that required addition of 100 μl lysis buffer (10 mM Tris-HCl {pH 8.3}, 50 mM KCl, 100 μg of gelatin, 0.45% Tween 20, 0.45% Nonidet P-40, 60 μg of proteinase K per ml), and lysing for 90 minutes at 56°C, followed by incubation at 95°C for 20 minutes to inactivate the proteinase K, all performed in a single tube to minimize handling [4]. Each tube with lysed sample was then spun at 1000g for 7 minutes to force the filter paper disc and other debris to the bottom of the tube and supernatants containing lysed samples were either immediately used in PCR or stored at -20°C for later use. A volume of 2 μl of lysate from FP-DBS was used in 50 μl PCR reaction for all studies unless indicated. To validate the assay, the amplified product from one PCR was verified by sequence analysis as being the desired sequence (not shown).

For testing of samples by HIV-1 *pol *real-time PCR to verify HIV-1 copy numbers, nucleic acids were extracted from the FP-DBS using standard Qiagen DNA extraction kit.

### FP-DBS samples

For initial studies FP-DBSs with known quantities of HIV-infected cells were made by spotting approximately 50 μl HIV negative blood along with ACH2 cells, which contain a single integrated copy of HIV-1 proviral DNA per cell [16,17], on S&S 903 filter paper (Schleicher & Schuell, Keene, NH). HIV negative blood spots were made from drops of blood with no anticoagulant mimicking blood collected from infant heel prick, and allowed to air-dry overnight. HIV-1 infected ACH2 cells were counted on a hemacytometer and the cells were diluted in sterile PBS to obtain the final expected 10, 5, 2 or 1 infected cells per 2 μl of eluate, which was the volume used for each PCR reaction. The quantified cells in PBS (10 μl) were then spotted onto 8mm punch of the FP-DBS prepared from HIV-negative blood, allowed to soak in and air-dry. In this case, the total number of cells changed very little from sample to sample, since the added ACH2 cells would represent a very small fraction of the total cells in a dried blood spot. To confirm the quantity of viral copies in the diluted HIV-1 infected ACH-2 cell suspension, at a later date, the DNA was extracted using the Qiagen DNA extraction kit, and HIV copy number was quantified using HIV-1 *pol *real-time PCR [14,15].

Archived FP-DBS, collected on S&S 903 filter paper (Schleicher & Schuell, Keene, NH) from 115 infants from Nairobi, Kenya of known infection status (56 HIV-1 positive and 59 HIV-1 negative), were collected, air-dried and shipped to Seattle. These archived FP-DBS that had been stored at ambient room temperature for 3 to 7 years in envelopes, in the Seattle laboratory with no desiccant were tested with the HIV-1 *pol *PCR FP-DBS assay. The operator was blinded to the infection status of the infant when the HIV-1 *pol *PCR FP-DBS assay was performed.

Subsequently, FP-DBS (N = 186) from more recent samples collected on S&S 903 filter paper (Schleicher & Schuell, Keene, NH) between the years 2007 to 2009 from infants aged < 1 year were tested on site in Nairobi, Kenya. The FP-DBS were prepared by spotting 50 μl of whole blood in EDTA, air-dried overnight and stored in zip-lock bags with a desiccant at ambient room temperatures before use, which was within one month of storage.

The infant blood samples for the FP-DBS were obtained as part of NIH-funded research studies with consent from the mothers or the caregivers of the infant, and tested for HIV infection with ethical approval from the Institutional Review board at University of Washington (approval # 98-7407-A) and Fred Hutchinson Cancer Research Center, USA (# 6341), and Kenyatta National Hospital, Kenya (approval # P4/01/2006).

## Results

In initial studies, various amounts of lysate were tested in the HIV-1 *pol *FP-DBS PCR to determine if there was inhibition due to heme and other factors in the lysed sample. As we had seen previously with the nested HIV-1 *gag *FP-DBS PCR assay [4], addition of 5 μl and greater of the FP-DBS lysate into a 50 μl PCR resulted in some inhibition of the reaction (not shown). In other studies, we found this was true for blood collected in no anticoagulant or with EDTA or ACD; the inhibition was even more pronounced using blood collected in heparin (data not shown). For this reason, we used 2 μl of lysate from FP-DBS for the HIV-1 *pol *FP-DBS PCR from FP-DBS prepared by spotting whole blood or blood collected in EDTA.

### Performance of HIV-1 *pol *PCR FP-DBS assay with spiked FP-DBS prepared from known HIV-1 copy numbers

To examine the ability of this 'in-house' HIV-1 *pol *PCR assay to detect the range of HIV-1 copy numbers from FP-DBS samples, defined quantities of ACH-2 cells were applied to FP-DBS. For this purpose, ACH2 cells were counted two different times (A & B) on two separate days (D1 & D2), as shown in Table 1. The total amount added to the FP-DBS was calculated so that 2 μl of the final lysate would be expected to have 10, 5, 2 and 1 HIV-1 proviral copies. To verify these numbers in parallel, extracted DNA from an aliquot of each tube of manually counted ACH2 cells was tested in triplicate with the HIV-1 *pol *real-time PCR assay [14,15], and the results from this assay gave an expected HIV-1 copy number that was within 2-fold of that predicted by cell count in 14 of 16 cases; the 2 discrepant cases were at the lowest cell count (Table 1). A total of 40 PCRs were performed on lysate from each cell preparation: in each case, four FP-DBSs were prepared and 10 HIV-1 *pol *PCRs were performed from every FP-DBS lysate (Table 1).

**Table 1:** Summary of HIV-1 *pol *FP-DBS PCR assay performed on DBS spiked with low copy number ACH2 cells

Cell count Copies/ml	pol real-time copies/rx (on ACH2 used to spike) avg 3 tests	No. positive (%) qualitative PCR on DBS lysate	Avg % D1 or D2	Avg % both days (D1 and D2)
10(A)D1	11.4	40(100)	98.7	98.1
10(B)D1	6.5	39(97.5)		
10(A)D2	17.5	38(95)	97.5	
10(B)D2	12.5	40(100)		
				
				
5(A)D1	12.6	26(65)	72.5	
5(B)D1	4.95	32(80)		72.5
5(A)D2	3.5	25(62.5)	72.5	
5(B)D2	3.6	33(82.5)		
				
				
2(A)D1	1.1	27(67.5)	75	46.9
2(B)D1	3.05	33(82.5)		
2(A)D2	1.5	11(27.5)	18.8	
2(B)D2	1.3	4(10)		
				
				
1(A)D1	1.6	18 (45)	56.2	38.7
1(B)D1	0.8	27(67.5)		
1(A)D2	0.1	9(22.5)	21.2	
1(B)D2	3.3	8(20)		

A total of 40 HIV-1 *pol *PCR assays were performed on FP-DBS spiked in duplicate (A and B) with known copies of HIV-1 in ACH-2 cells (manually counted and quantified by HIV-1 *pol *real-time PCR assay done) on two different days (D1 and D2). The results of the HIV-1 *pol *PCR assay are indicated as an average percentage of HIV-1 proviral copies detected from the spiked FP-DBS.

The results of HIV-1 *pol *PCR FP-DBS assay showed that one HIV-1 proviral copy was detectable in 38.7% of tests (160 total tests), 2 copies in 46.9% of tests, 5 copies in 72.5% of tests, and 10 copies in 98.1% of tests, as expected based on Poisson distribution (Table 1). This experiment was repeated a second time on two separate days with similar results (data not shown). Comparable results were also obtained in smaller studies using FP-DBS prepared from PBMCs infected with different HIV subtypes (A, C and D; data not shown), which is consistent with our previous studies showing that with the same primers, HIV-1 *pol *PCR assay in the real-time format can detect these HIV subtypes [15]. Overall, these data with unpurified cell lysates using HIV-1 *pol *PCR FP-DBS assay compared favourably with results of the real-time HIV-1 *pol *PCR assay where nucleic acid was prepared using a Qiagen purification method prior to PCR [14]. These data suggest that the HIV-1 *pol *PCR FP-DBS assay is able to reliably detect as low as a single copy of HIV provirus from a FP-DBS with minimal nucleic acid purification.

### *pol *PCR FP-DBS assay on stored samples from infants of known HIV-1 infection status

We next evaluated the HIV-1 *pol *PCR FP-DBS assay using stored FP-DBS from infants of known infection status, as defined by prior testing of two sequential samples with the HIV-1 *gag *PCR assay [4,18]. These FP-DBS had been prepared by spotting whole blood on the filter paper, air-dried and stored at ambient room temperature for several years. One hundred and fifteen FP-DBS (56 HIV-1 positive and 59 HIV-1 negative) were tested with the operator blinded to the infection status. The lysate was tested in parallel with the two round, nested HIV-1 *gag *PCR that was used previously and the single round HIV-1 *pol *PCR FP-DBS assay. The nested HIV-1 *gag *PCR that amplified a 142bp of fragment, nearly the same size fragment as the single round HIV-1 *pol *PCR FP-DBS assay served as a control for the integrity of the FP-DBS samples, which had been stored at ambient room temperature for several years.

The repeat testing of the stored FP-DBS from these infants with the original HIV-1 *gag *PCR showed 95.6% agreement with the prior testing that established infection status, suggesting that the long-term storage had not significantly compromised the samples. These same samples were tested with the HIV-1 *pol *PCR FP-DBS assay. The sensitivity and specificity of the HIV-1 *pol *PCR assay in relation to the known HIV-1 infection status of the infants was 92.8% and 98.3% respectively (Table 2).

**Table 2:** A 2 × 2 table for performance of HIV-1 *pol *FP-DBS PCR assay on archived FP-DBS samples

		KNOWN HIV-1 STATUS		
		
		Positive	Negative	TOTAL
***pol *PCR HIV-1 results**	**Positive**	52	1	53
	
	**Negative**	4	58	62

	**TOTAL**	56	59	115

Samples were obtained from infants of known HIV-1 infection status (56 HIV-1 positive and 59 HIV-1 negative). The sensitivity of HIV-1 *pol *PCR FP-DBS assay on archived FP-DBS was 92.8% and specificity of 98.3%.

### Comparison of *pol *PCR FP-DBS assay with commercial DBS-FP assay

**Figure 1:**
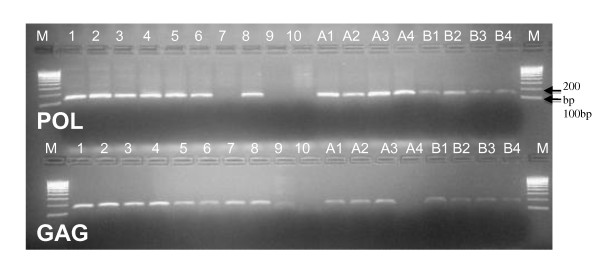
**Gel picture of amplified products using 'in-house' HIV-1 *gag *and *pol *FP-DBS PCR assay**. Gel picture of the HIV-1 *pol *(top panel - 160bp) and *gag *(lower panel - 152bp) PCR products. Lanes labeled from 1- 10 are PCR products from the ACH copy number controls: 1-2 are 100 HIV copies; 3-4 are 10 copies; 4-8 are 2 copies; 9 and 10 are negative controls using DNA from an uninfected T cell cell-line and water as the PCR template, respectively. PCR results from two samples of DBS-FP from infants who tested positive with the Roche Amplicor assay are labeled as A1-A4 and B1-B4, with 1-4 indicating quadruplicate tests. Based on the results show, both infants would be defined a HIV-1 DNA PCR positive according to testing algorithm for the' in-house' *gag *and *pol *FP-DBS FP PCR. The first and last lane in the gel picture (labeled M) in both the panels is the molecular weight marker - Hyperladder IV.

The assay was transferred from the Seattle-based laboratory to a newly established molecular virology laboratory at the University of Nairobi. This laboratory included a PCR set-up room that had established standard practices to minimize the potential for introduction of PCR product, plasmid and other possible PCR contaminants. In this laboratory, we tested follow-up FP-DBS samples prepared from whole blood in EDTA collected from infants that were initially reported as HIV-1 positive using the Roche Amplicor v1.5 assay at the National Reference Laboratory for Early Infant Diagnosis testing at Kenya Medical Research Institute or at Kenyatta National Hospital, in Nairobi, Kenya, typically within the prior month. For this confirmatory HIV-1 testing, the infant was rebled and a fresh FP-DBS was prepared from 50 μl of whole blood, air-dried and lysed for PCR analysis. The lysate was then tested in quadruplicate PCRs using both the HIV-1 *gag *and *pol *PCRs (see gel picture Figure 1).

As a control, we also included randomly within the test runs, 25 FP-DBS collected from HIV-1 seronegative adults, as defined by two parallel rapid HIV-1 serological assays. The test operator was blinded to HIV-1 serostatus of the control samples and upon testing all the quadruplicate PCR tests on the samples, both for *gag *and *pol *products were negative.

**Figure 2:**
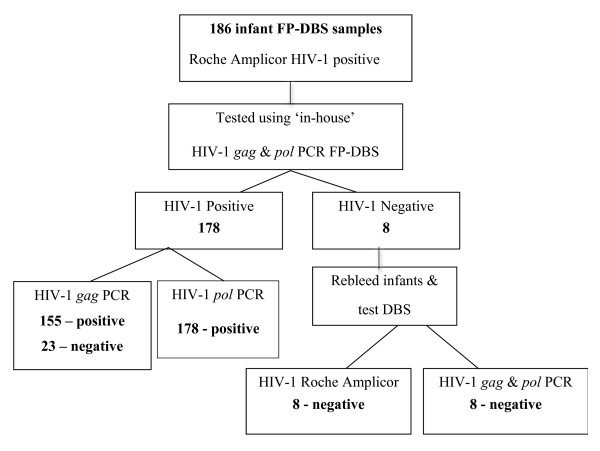
**Experimental approach for performance of HIV-1 *gag *and *pol *FP-DBS PCR assay for detection of HIV-1 infection from FP-DBS obtained from HIV-1 positive infants as defined by Roche Amplicor v1.5 assay**. 186 recently collected FP-DBS that were determined in the prior ~month to be HIV-1 positive by Roche Amplicor v1.5 assay were tested by HIV-1 *gag *and *pol *PCR assays. Of these 178 FP-DBS from the infants were identified as HIV-1 positive. Retesting of subsequent blood samples from the 8 discordant infants showed that they were HIV-1 negative. Thus, the sensitivity of HIV-1 *pol *PCR assay was found to be 100% (178 of 178) for detection of confirmed HIV-1 positive infants from FP-DBS.

Of the 186 HIV-1 positive samples from the infants that were defined as positive with Roche Amplicor^® ^HIV-1 DNA assay version 1.5, 178 samples were confirmed as positive by HIV-1 *pol *PCR FP-DBS assay (Figure 2). Of the 8 samples that were negative by both HIV-1 *pol *and *gag *PCRs, the infants were re-bled and re-tested using all the tests, the Roche Amplicor^® ^HIV-1 DNA v1.5 assay (at the reference lab) and the HIV-1 *gag *and *pol *PCR FP-DBS assay. Upon retesting all of the 8 infants were identified as HIV-1 negative by all assays, suggesting the initial results using the commercial Roche Amplicor^® ^assay were false positive results. However, we could not determine whether the initial results of the Roche Amplicor^® ^assay for the 8 infants were the result of false positive tests or whether sample mix-up could have contributed to these results. The HIV- gag PCR showed only 87% sensitivity (155 of 178 positives) compared to both the Roche Amplicor^® ^and HIV-1 *pol *PCR FP-DBS assay.

The infants were all under the age of 12 months. Most (46%) were between 3 and 6 months, but we also sampled younger infants (25% < 3 months) and older infants (29% > 6 months), as shown on Table 3. Of the 178 HIV-1 positive infants as determined by DNA PCR on FP-DBS using all the 3 methods (Roche Amplicor^® ^and 'in-house' *gag *and *pol *assay), 110 infants had HIV-1 subtype data available based on *pol *sequence. The majority (69%) of the infants were identified as infected with HIV-1 subtype A, and others with subtype D (23%), subtype C (7%) and intersubtype recombinant AD (1%) (Table 4). This is very similar to the subtype distribution in Nairobi [19].

**Table 3:** Age demographics of infants tested for HIV-1 DNA PCR from freshly collected DBS samples in Nairobi, Kenya

	Age of infants (months)				
	
	< 3	3 to < 6	6 to < 9	9 to < 12	Total
**No. infants**	47	85	39	15	186
	25.3%	45.7%	21.0%	8.0%	

Mean Age	4.6 months				
Range	3 wks - 11.5 months				

**Table 4:** HIV-1 subtypes in infants who tested positive with the HIV-1 pol FP-DBS PCR assay in Nairobi, Kenya

HIV-1 subtypes				
**A**	**D**	**C**	**AD recomb**	**Total**

76	25	8	1	110
69%	23%	7%	1%	

Note: HIV-1 subtypes based on 667bp *polymerase *genome

HIV-1 subtypes available for only 110 of the 178 HIV-1 positive DBS-FP from infants in the Nairobi study.

Overall, from the 178 samples confirmed positive with Roche Amplicor^® ^assay, all were positive by HIV-1 *pol *PCR, indicating that the sensitivity and specificity of the HIV-1 *pol *PCR assay was 100% in this study. As this is based on using data from 4 HIV-1 *pol *PCR tests to detection infection, we also examined the sensitivity if we considered the results from just the first (single test), the first two (duplicate) or the first 3 (triplicate) HIV-1 *pol *PCR tests. Based on our results of HIV-1 *pol *PCR assay the likelihood of missing true positives if we had performed the assay singly would be 14.6% (26 of 178), in duplicate testing would be 6.7% (12/178), in triplicate testing would be approximately 0.6% (1/178), and on quadruplicate testing would be none.

## Discussion

In summary, we describe here a sensitive HIV-1 *pol *PCR FP-DBS assay for detection of infant infection. The advantages of this assay include the fact that it requires minimal manipulation of the sample compared to assays that rely on extraction of nucleic acids and nested PCR methods. This method can detect a single copy of HIV provirus and has been validated on HIV-1 sequences of multiple subtypes.

The HIV-1 *pol *PCR FP-DBS assay was compared to results from historical studies in Seattle as well as samples from infants who recently tested positive by the Roche Amplicor^® ^HIV-1 DNA Test, version 1.5 assay in Nairobi. The sensitivity and specificity of the HIV-1 *pol *PCR FP-DBS assay was > 90% on archived samples stored for more than 3 years at room temperature. The combined sensitivity of the HIV-1 *pol *PCR FP-DB assay using the archived (N = 56 positive) and recent samples (N = 178 positive) was 98.3% (Table 5). These comparisons are based on quadruplicate testing, which maximizes detection of low HIV copies in a sample. Using this approach, we detected 100% of confirmed positive samples from infants recently identified as HIV positive by the Roche Amplicor^® ^HIV-1 DNA assay. Based on initial field site testing of FP-DBS from 178 HIV positive infants, this HIV-1 *pol *PCR FP-DBS assay with a single, duplicate or triplicate PCR testing would be predicted to detected ~85%, 93% and 99% of HIV-1 positive samples, respectively.

**Table 5:** A 2 × 2 table for performance of HIV-1 *pol *FP-DBS PCR assay on all FP-DBS

		KNOWN HIV-1 STATUS		
		
		Positive	Negative	TOTAL
***pol *PCR HIV-1 results**	**Positive**	230	1	231
	
	**Negative**	4	91	95

	**TOTAL**	*234	^$^92	326

A total of 115 archived and 211 current FP-DBS samples of known HIV-1 infection status were tested. Overall the sensitivity of HIV-1 *pol *PCR FP-DBS assay on all FP-DBS samples (archived and recent samples) was 98.3% and specificity of 98.9%.

* a total of 56 archived FP-DBS samples and 178 recent FP-DBS samples obtained from infants.

^$ ^a total of 59 archived and 33 recent FP-DBS samples (8 obtained from infants and 25 from adults).

The HIV-1 *pol *PCR FP-DBS assay samples a lower total volume of blood than the commercial assays that go through a purification step to remove inhibitors, which lengthens and complicates these assays. Thus, while the Roche Amplicor^® ^HIV-1 DNA assay typically tests for HIV in ~12.5 μl of blood eluate (assuming processing a 50 μl blood spot by adding 200 μl lysate and testing 50 μl), the HIV-1 *pol *PCR FP-DBS assay only tests 0.2-0.3 μl blood eluate per PCR (in this assay only ~a quarter of the blood spot is sampled, ~10-12 μl of blood); this is lysed in 100 μl and 2 μl of lysate is used per PCR. However, this lower blood volume used in the HIV-1 *pol *PCR FP-DBS assay should be adequate to sample HIV-infected cells in an infant sample. Infant blood contains an estimated 75.4 ± 104.3 HIV proviral copies per 1000 PBMC [20]. Assuming that 1 μl of blood contains 1 million total cells, of which 5000 are PBMCs [21], then there are ~1000 PBMC sampled in each PCR. Thus, each PCR that includes 0.2 μl of infant blood typically contains multiple HIV copies that can be detected in this assay that should be amplified with this single HIV-1 copy detection PCR method.

Even when performed in quadruplicate, this qualitative DNA assay is economical and costs just a few dollars per patient, compared to some of the commercial assays that are nearly 5 to 10 times more expensive (~20-50 US$). While quadruplicate testing may provide optimal sensitivity, this assay may also be highly sensitive and specific when PCRs are performed in duplicate or triplicate. However, quadruplicate testing may increase the ability of this assay to detect infants that are destined to become slow progressors, who are estimated to have lower HIV proviral copy numbers (11.8+18.8 HIV copies/1000 PBMC; [20]). Laboratories that adapt this assay may wish to compare the performance of quadruplicate testing versus using fewer replicate tests to determine the number of PCR tests that are optimal to suit their needs.

Importantly, when transferred to a new laboratory in Nairobi for on-site testing in a setting that applied stringent measures to minimize PCR contamination, the assay showed very high sensitivity and specificity compared to the results of commercial assays from established reference laboratories in the region. Of course, the performance of this assay in other settings may vary depending on the established protocols and expertise of the laboratory, as is true with the use of any PCR-based assays-either 'in-house' or commercial. One limitation of the study results described here is that they focused primarily on samples from infants recently detected as HIV-1 positive by the Roche Amplicor^® ^HIV-1 DNA Test. Further studies of infants born to HIV-positive mothers that are not pre-screened in this manner will be needed to more precisely define the sensitivity and specificity of this assay in a clinical setting in real time. In this case, it will be important to not only verify the findings with a second assay, but also to test a follow-up sample from each infant because other assays also have limitations in their performance, such as the false-positive results using the Roche Amplicor^® ^HIV-1 DNA Test described here. While further testing by other laboratories will be useful for validating the performance of this assay, these findings suggest that the HIV-1 *pol *PCR FP-DBS assay provides a reliable and rapid method, and is an economical assay for early detection of HIV-1 infection in infants.

## Conclusions

There is an urgent need for an economical and reliable assay for early HIV-1 infant diagnosis, especially for low-resource countries. This study has validated an economical 'in-house' HIV-1 *pol *PCR FP-DBS assay that is highly sensitive and specific when compared to a commercial Roche Amplicor^® ^v1.5 FP-DBS assay. This study highlights the need for potential adaptation of this qualitative DNA-based assay in development of a rapid point-of care diagnostics assay for early infant HIV-1 diagnosis. This single round *pol *PCR FP-DBS can therefore be a useful tool for early infant HIV-1 diagnosis in Africa especially where the HIV epidemic prevails and resources are limited.

## Competing interests

The authors declare that they have no competing interests.

## Authors' contributions

BHC helped design aspects of the study, validated and performed assays, analyzed the data and helped draft the manuscript. SE validated and performed assays, and provided input into the manuscript. MM and MN participated in the study by performing the assay on the samples in the field. SF was helpful in setting up the molecular virology laboratory in the field and assisting in transferring the technology and training staff on the methods. GJ-S and DW as Principal Investigators of the research project from which clinical samples were obtained, provided the samples in the field for evaluation of the assay and gave input into the study design. JO conceived the idea and led the study design, implementation of the program and drafting and editing of the manuscript. All authors contributed to the data analysis and read and approved the final manuscript.

## Pre-publication history

The pre-publication history for this paper can be accessed here:

http://www.biomedcentral.com/1471-2431/11/18/prepub
